# Fiber mixtures containing chicory inulin, wheat dextrin, and cellulose, or tapioca dextrin alone, beneficially modulate microbial metabolic activity and composition in short-term colonic simulations

**DOI:** 10.3389/fnut.2026.1749272

**Published:** 2026-02-18

**Authors:** Jonas Ghyselinck, Lynn Verstrepen, Cindy Duysburgh, Regina Wiche, Katharina S. Kuhn, Marianne Müller, Valentin Faerber-Frottier, Massimo Marzorati

**Affiliations:** 1 ProDigest, Zwijnaarde, Belgium; 2 Fresenius-Kabi Deutschland GmbH, Bad Homburg, Germany; 3 CMET, University of Ghent, Gent, Belgium

**Keywords:** cellulose, dietary fiber, enteral nutrition, inulin, prebiotic, short-chain fatty acids, tapioca dextrin, wheat dextrin

## Abstract

**Background:**

Dietary fiber blends are commonly included in enteral nutrition to support gut health, a key component of Medical Nutrition Therapy. These blends help maintain intestinal integrity and promote a healthy gut microbiota. In this *in vitro* study, we investigated the prebiotic potential of three combinations of inulin, wheat dextrin, and cellulose, as well as pure tapioca dextrin.

**Methods:**

Predigested test products were incubated with fecal suspensions from three healthy donors in a colonic simulation model. Microbial fermentative activity, metabolite production, and composition were analyzed at 0, 6, 24, and 48 h. After 48 h, samples were tested for their effects on intestinal barrier function and cytokine production in a Caco-2/Tohoku Hospital Pediatrics-1 (THP1) co-culture model.

**Results:**

All test products were well fermented, resulting in a significant increase in total short-chain fatty acid (SCFA) production at 48 h (*p* < 0.05). Individual SCFA levels varied among donors and according to fiber composition. Ammonium levels, which indicate proteolytic fermentation, were significantly decreased. All fiber mixtures and tapioca dextrin increased the abundance of Bacteroidetes, Actinobacteria (including *Bifidobacterium*), and Firmicutes. Improvements in intestinal barrier integrity and immunomodulatory effects were donor-dependent, with the strongest effects observed in Donor C. Notably, fiber mixtures with lower or no cellulose statistically significantly increased anti-inflammatory interleukin-10 (IL-10) levels across all donors.

**Conclusion:**

Dietary fiber mixtures containing inulin, wheat dextrin, and cellulose, as well as pure tapioca dextrin, demonstrated prebiotic effects and reduced potentially toxic products of proteolytic fermentation. These findings indicate that such fiber mixtures in enteral nutrition may support gut integrity and modulate immune modulation, benefiting patients receiving Medical Nutrition Therapy.

## 1 Introduction

The gut microbiome plays a pivotal role in human health and disease (1, 2). More than 50 bacterial phyla colonize the gut, with a predominance of Gram-positive Firmicutes and Gram-negative Bacteroidetes (1, 2). Dietary intake profoundly influences the composition and metabolic activity of the gut microbiota, particularly in individuals receiving Medical Nutrition Therapy, such as enteral nutrition via tube feeding or oral nutritional supplements, commonly applied in conditions including critical illness, cancer, chronic kidney disease, gastrointestinal disorders, or post-surgical recovery (3–6). These conditions often lead to gut dysbiosis, characterized by reduced microbial diversity, loss of beneficial saccharolytic bacteria that ferment carbohydrates to short-chain fatty acids (SCFAs) (e.g., *Bifidobacterium*), and an overgrowth of proteolytic bacteria (e.g., *Bacteroides*), which produce harmful by-products like ammonia and toxins (2, 7–10). Deleterious consequences of gut dysbiosis include impaired intestinal barrier integrity, increased permeability, and dysregulated immune and inflammatory responses (5, 7, 11).

To address these challenges, many enteral nutrition formulations include dietary fiber blends to promote gut homeostasis and modulate immunity (7). Dietary fiber, as defined in EU Directive 2008/100/EC (12), is an edible carbohydrate polymer with three or more monomeric units that are neither digested nor absorbed in the small intestine. They must confer beneficial physiological effects, such as fermentability by colonic microbiota and/or improvements in metabolic health. The term “prebiotic” is often used synonymously with soluble dietary fiber. Similar to dietary fibers, prebiotics must resist digestion. However, they must also undergo saccharolytic fermentation by intestinal microbiota, and promote bacterial growth or activity to support host health (13, 14).

Dietary fibers, like inulin, are fermented by intestinal bacteria (e.g., *Bifidobacterium*), producing SCFAs such as acetate, propionate, and butyrate. These SCFAs contribute to gut health by reducing inflammation, strengthening the intestinal barrier, and supporting immune regulation (15–20). Bacterial cross-feeding—the reutilization of fermentation products produced by one bacterial strain by another—can considerably increase the efficiency of SCFA production (14, 20–22). SCFAs, especially butyrate, serve as an important energy source for colonocytes during intestinal absorption. Alternatively, they are transported to the liver, where propionate is mainly involved in gluconeogenesis, and acetate and butyrate are mainly involved in lipid synthesis pathways (14, 20). SCFAs impart known benefits to overall gut health (23–25). They are associated with reduced intestinal inflammation and improved intestinal barrier integrity (25), which may protect against “leaky gut” (26). Additionally, they are reported to play a role in immune system function and the regulation of inflammatory responses (22).

Enteral formulations often include fiber blends, such as inulin (a non-digestible fructan sourced from chicory root), wheat dextrin (a non-digestible dextrin refined from wheat starch), and tapioca dextrin (a non-digestible dextrin derived from tapioca starch), with reported prebiotic properties (27–29). Moreover, cellulose, an insoluble fiber, is often included due to its bulk-forming properties that support normalization of bowel movements (30). Although the prebiotic effects of individual fibers are well documented, the impact of specific fiber combinations and varying proportions remains unclear. This study aimed to assess the impact of fiber mixtures with different proportions of inulin, wheat dextrin, and cellulose, as well as tapioca dextrin alone (i.e., as a single fiber source comparison), on the gut microbiota. Using an established *in vitro* colonic simulation model, we evaluated fermentative activity, metabolite production, microbial community composition, and effects on inflammation-induced intestinal barrier disruption and cytokine production in a healthy population, as dietary fiber recommendations have mainly been put forward for this group. Nevertheless, these recommendations are equally important for specific patient populations, such as individuals at risk for malnutrition. Therefore, the objectives of the current study are highly relevant for understanding the design of enteral nutrition formulations intended to support gut health and immune function in patients requiring Medical Nutrition Therapy.

## 2 Materials and methods

### 2.1 Sample collection and experimental procedures

Fecal samples were collected from three healthy donors—acknowledging that this sample size may still be considered limited for capturing interindividual variability—aged between 25 and 45 years who consumed a mixed Western diet, had no history of chronic diseases, and had no antibiotic use during the 6 months preceding the experiment. Fecal suspensions were prepared, mixed with a cryoprotectant (31), aliquoted, flash-frozen, and stored at −80 °C until needed. Effects of the test products on the gut microbiota were evaluated relative to a fiber-free control containing only the fecal microbiota of a given donor and a background nutritional medium. The collection and use of the fecal samples were performed in accordance with the protocol approved by the Ethics Committee of the University Hospital Ghent (reference number: ONZ-2022-0267; approved on 29 July 2022). Informed consent was obtained from all subjects involved in the study.

### 2.2 Test products

Fiber mixtures evaluated in this study contained chicory inulin, wheat dextrin, and cellulose in varying proportions: (a) Fiber mixture 1: chicory inulin 43.8%, wheat dextrin 18.8%, cellulose 37.5%; (b) Fiber mixture 2: chicory inulin 35.0%, wheat dextrin 45.0%, cellulose 20.0%; and (c) Fiber mixture 3: chicory inulin 83.3%, wheat dextrin 16.7%. The mixtures are used as fiber sources in tube feeds and oral nutritional supplements (Fresubin® formula by Fresenius Kabi), which are designed as food for special medical purposes for patients with or at risk of malnutrition. Additionally, the prebiotic tapioca dextrin was evaluated as a single fiber source. All test products were provided by Fresenius Kabi, Bad Homburg, Germany.

### 2.3 Predigestion

A 40 g/L stock solution of each test product was prepared and exposed to conditions that simulate oral, gastric, and small intestinal passage (32). Following this, the solutions were placed inside 0.5 kDa dialysis membranes, which were then sealed. The solutions were dialyzed in 3.75 g/L NaHCO_3_, pH 7.0, for 24 h to remove monosaccharides and disaccharides from the predigested solutions, retaining the non-digestible fibers. Additionally, a predigestion blank medium was generated in parallel to be added to the fiber-free controls in the colonic simulations. This was achieved by running each step of the predigestion (including dialysis) in the absence of the test products.

### 2.4 Short-term colonic incubations

Each reactor was first filled with 56 mL of nutritional medium (PD01; ProDigest, Gent, Belgium). Then, 7 mL (10% [v/v]) of predigested/dialyzed test product or predigestion-conditioned blank medium was added to each reactor, followed by 7 mL (10% [v/v]) of freshly prepared fecal inoculum from one of three healthy human donors. The total reactor volume was 70 mL, with a starting test product concentration of 4 g/L (assuming no absorption). The reactors were incubated at 37 °C with continuous shaking (90 rpm) in an anaerobic atmosphere for 48 h. Each condition was tested in duplicate.

### 2.5 Microbial metabolic activity: pH, gas production, short-chain fatty acids, and ammonium

At 0, 6, 24, and 48 h, changes in pH, gas pressure, SCFAs, branched chain fatty acids (BCFA), and lactate were measured; ammonium levels were determined at 0, 24, and 48 h. Changes in pH were measured with a Senseline F410 pH meter (ProSense, Oosterhout, The Netherlands), and gas production was monitored using a handheld pressure indicator (CPH6200; Wika, Echt, The Netherlands). The methods of De Weirdt et al. were used to measure acetate, propionate, and butyrate, and BCFAs (isobutyrate, isovalerate, and isocaproate) (33). Lactate levels were evaluated using an enzymatic assay kit from R-Biopharm (Darmstadt, Germany) according to the manufacturer’s instructions. Ammonium levels were assessed using the method of Tzollas et al. (34).

### 2.6 DNA extraction and 16S RNA sequencing

Total DNA was isolated as described by Duysburgh et al. (35). 16S-targeted Illumina sequencing utilized primers spanning two hypervariable regions (V3–V4) of the 16S ribosomal RNA (rRNA) gene (341F, 5′-CCTACGGGNGGCWGCAG-3′; 785R, 5′-GACTACHVGGGTATCTAAKCC-3′) (36, 37). Sequencing of 2 × 250 bp (paired sequencing) yielded 424-bp amplicons (LGC Genomics GmbH, Berlin, Germany). Amplicons of this size are taxonomically more useful than smaller fragments. Read assembly and cleanup were performed according to the MiSeq SOP (Schloss lab) (37, 38). Briefly, reads were assembled into contigs, and alignment-based quality filtering was performed (alignment to the mothur-reconstructed SILVA SEED alignment, version 138.0). Chimeras were then removed (vsearch version 2.13.3), and a naïve Bayesian classifier (39) and SILVA NR version 138_1 were used to assign taxonomy. Contigs were clustered into Operational Taxonomic Units (OTUs) at 97% sequence similarity using mothur (version 1.44.3) (OTU-based clustering was used instead of amplicon sequence variant (ASV)-based approaches (e.g., deficiency of adenosine deaminase 2 [DADA2]) as part of prior validated workflows). Sequences that were classified as Archaea, Eukaryota, mitochondria, or chloroplasts, or those that could not be classified, were removed. The representative sequence was defined as the most abundant sequence within an OTU. Reads with a maximum abundance ≤5 across samples were removed as they were considered to be bacteria with low biological impact or artefacts.

### 2.7 Microbial community analysis by quantitative PCR

The abundances of the two dominant colonic bacterial phyla, Firmicutes and Bacteroidetes, and two specific groups of interest (due to their links to health-promoting effects), *Bifidobacterium* and *Akkermansia muciniphila*, were monitored by quantitative PCR (qPCR). qPCR for Firmicutes and Bacteroidetes was performed as previously described by Guo et al. (40). The method reported by Collado et al. was used to quantify *A. muciniphila* (41), and the method reported by Rinttilä et al. was used to quantify *Bifidobacterium* spp. (42).

### 2.8 Flow cytometry

To determine the total number of bacterial cells, samples were analyzed by flow cytometry using a BD Accuri C6 Plus Flow Cytometer (BD Biosciences, Franklin Lakes, NJ, USA) set to the high flow rate. Bacterial cells were separated from medium debris and signal noise by applying a threshold level of 700 on the SYTO channel. Parent and daughter gates were set to determine the populations. Using these cell counts, metagenomics data on relative abundance were converted to absolute abundance by multiplying each sample’s relative abundance by the total cell count, as previously reported by Vandeputte et al. (43).

### 2.9 Cell culture

Co-culture experiments were performed using Caco-2 (HTB-37; American Type Culture Collection) and phorbol-12-myristate-13-acetate (PMA) differentiated THP1-Blue™ cells (InvivoGen, San Diego, CA, USA) as previously described (44, 45). First, Caco-2 cells were seeded in 24-well semi-permeable inserts and cultured for 14 days. THP1-Blue™ cells were seeded in 24-well plates and treated with PMA to induce differentiation. Subsequently, Caco-2-bearing inserts were positioned onto the PMA-differentiated THP1-Blue™ cells for further experimentation, following previously established protocols (44). Briefly, sterile filtered (0.22 μm) colonic suspensions were added to the co-cultures. After 24 h of incubation, transepithelial electrical resistance (TEER) was measured in Caco-2 cells. Next, cells were stimulated with 500 ng/mL ultrapure lipopolysaccharide (LPS; *Escherichia coli* K12) (InvivoGen, San Diego, CA, USA) for 6 h. Supernatants were then collected and processed to assess nuclear factor kappa-light-chain-enhancer of activated B cells (NF-κB) activity and levels of select chemokines and cytokines. NF-κB activity was determined using the QUANTI-Blue reagent (InvivoGen, San Diego, CA, USA) to measure SEAP levels in basolateral supernatants according to the manufacturer’s instructions. Levels of human interleukin-1β (IL-1β), IL-6, IL-8, IL-10, tumor necrosis factor (TNF)-*α*, and C-X-C motif chemokine ligand 10 (CXCL10) in the co-culture basolateral supernatants were measured using Luminex® multiplex (ProcartaPlex immunoassays, ThermoFisher Scientific) according to the manufacturer’s instructions. Samples from the colonic incubations were obtained as biological duplicates and used in the cell co-culture assay as technical duplicates, resulting in quadruplicate values for each test condition.

### 2.10 Data processing and statistics

Total SCFA level was calculated by adding together the levels of acetate, propionate, and butyrate for each condition at each timepoint. For analysis of microbial metabolic activity, paired two-sided *t*-tests were used to determine significant differences between the test product and the fiber-free control.

Per donor and for each bacterial group, fold changes (FC; ratio of abundance at 6 or 24 h [T1] vs. abundance at start of incubation [T0]) were calculated for test products and fiber-free control. Treatments with fold changes that differed from fiber-free control across the donors were identified. A bacterial enrichment was considered significant if it exceeded the enrichment observed in the fiber-free control.

*α*-Diversity was evaluated using four methods: (a) observed taxa (measure for species richness), (b) Chao1 (measure for species richness), (c) Shannon (measure for species richness and evenness), and (d) Simpson (measure for species richness and evenness, giving more weight to common or dominant species). Paired two-sided *t*-tests were used to determine significant differences between test products and the fiber-free control. *β*-Diversity was assessed using Discriminant Analysis of Principal Components (DAPC), which joins two methods to assess effects on population structure. To accomplish this, sequence data were transformed using principal component analysis, and clusters were then identified using discriminant analysis (46), aiming to maximize among-group variation and minimize within-group variation.

Linear discriminant analysis effect size (LEfSe) (47) and treeclimbR (48) analyses were performed to identify the taxa most likely to explain differences between treatments. All features shown in the LEfSe plots met a criterion of *p* ≤ 0.05 for the Kruskal–Wallis and Wilcoxon tests; the 50 most significant features were included in the plot. No restrictions were put forward with respect to minimal linear discriminant analysis (LDA) scores, but, in general, LDA scores ≥ 2.0 were considered biologically relevant. The higher the LDA score, the higher the difference in abundance between the two test conditions. The results of the treeclimbR analysis are shown using volcano plots. The cut-off for statistical significance was set at *p* < 0.05 (or -log_10_[0.05] = 1.3 on the y-axis). Bacterial enrichments with a –log (*p*-value) > 1.3 were considered statistically significant. Taxa were classified into four different categories: (a) not significant and not biologically relevant (−2 < log_2_FC < +2, and −log_10_[*p*-value] < 1.3), (b) biologically relevant, but not statistically significant (log_2_FC < −2 or log_2_FC > +2, and −log_10_[*p*-value] < 1.3), (c) statistically significant, but not biologically relevant (−2 < log_2_FC < +2, and −log_10_[*p*-value] > 1.3), and (d) biologically and statistically significant (log_2_FC < −2 or log_2_FC > +2, and −log_10_[*p*-value] > 1.3).

To assess differences in TEER and immune markers between the test products and fiber-free control for individual donors, a two-way analysis of variance (ANOVA) with Dunnett’s multiple comparisons test against the fiber-free control was used. Cytokine data are shown normalized to the LPS control. To assess differences in TEER and immune markers between the test products and the fiber-free control, paired two-tailed *t*-tests were performed on the average of the three donors, using the average of the technical replicates for each donor as input values.

A *p*-value of < 0.05 was considered statistically significant. All statistical analyses were performed using GraphPad Prism version 9.5.0 for Windows (GraphPad Software, San Diego, CA, USA).

## 3 Results

### 3.1 Effects on microbial metabolic activity

**Figure 1:**
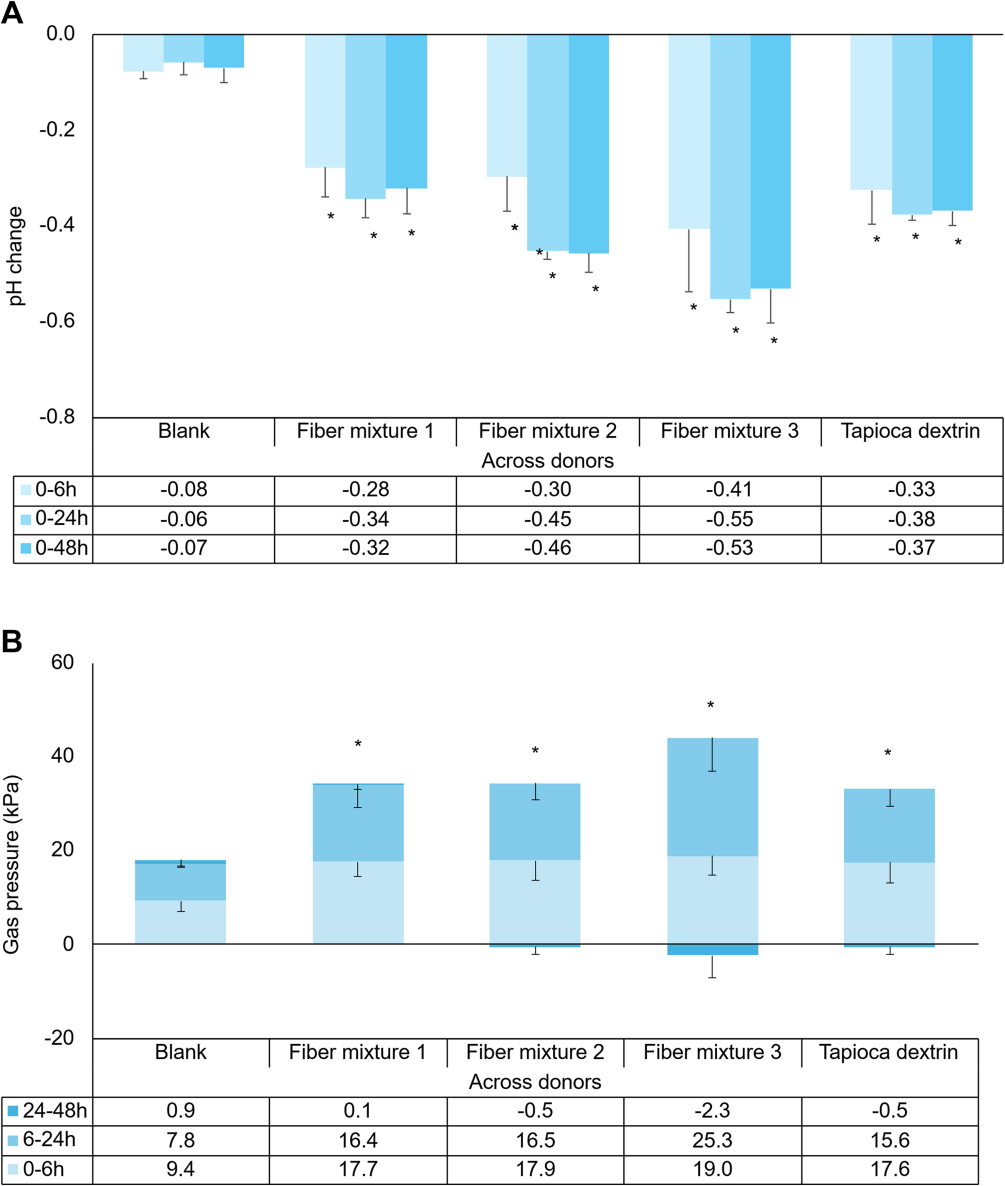
Effect of fiber mixtures and tapioca dextrin on **(A)** pH and **(B)** gas change at different time intervals. Each condition was tested in triplicate. Data for average values were derived using data from three healthy donors (means of biological replicates across donors). Error bars represent standard deviation. Two-sided *t*-tests were used to determine significant differences between each supplemented condition and the fiber-free control (blank) across the entire 48-h period. *p < 0.05.

General markers of microbial fermentation, pH decrease, and gas production were affected by the fiber mixtures and tapioca dextrin. pH decreased significantly starting from 6 h following test product supplementation, and gas production significantly increased relative to the fiber-free control in all test conditions (Figures 1A,B).

**Figure 2:**
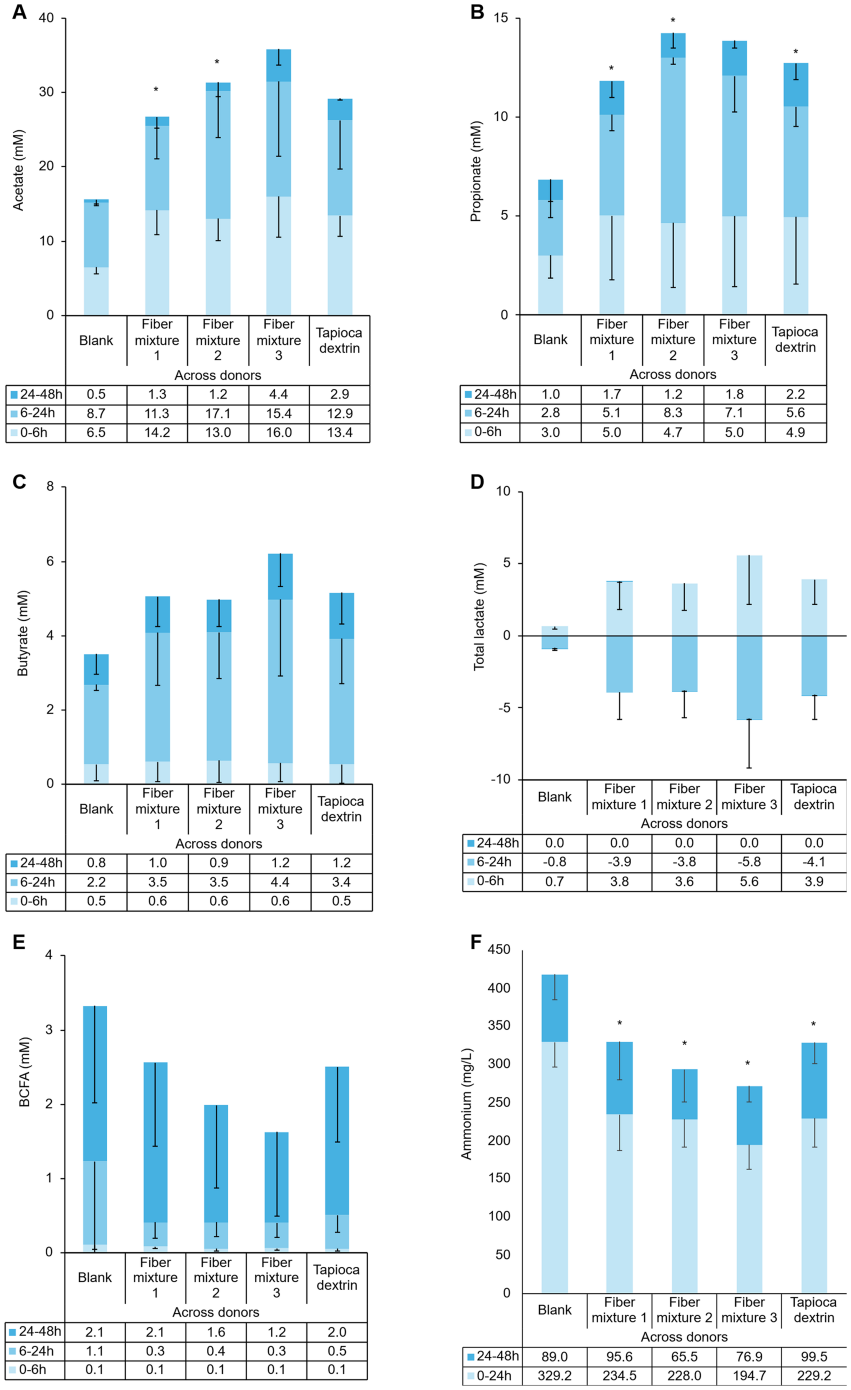
Effect of fiber mixtures and tapioca dextrin on **(A)** acetate (mM), **(B)** propionate (mM), **(C)** butyrate (mM), **(D)** lactate (mM), **(E)** branched chain fatty acids (BCFA; mM), and **(F)** ammonium (mg/L) at different time intervals. Each condition was tested in triplicate. Data for average values were derived using data from three healthy donors (means of biological replicates across donors). Error bars represent standard deviation. Two-sided *t-t*ests were used to determine significant differences between each supplemented condition and the fiber-free control (blank) across the entire 48 h period. *p < 0.05. BCFA, branched chain fatty acid.

Measures of saccharolytic fermentation (i.e., SCFA levels) demonstrated activity with the test products, as significant increases in total SCFA levels were observed for all test products when compared to the fiber-free control over the complete incubation period (FC = 1.7 with *p* = 0.0011, FC = 1.9 with *p* = 0.0109, FC = 2.2 with *p* = 0.0236, and FC = 1.8 with *p* = 0.0175 for fiber mixtures 1, 2, 3, and tapioca dextrin, respectively). Levels of acetate and propionate were significantly increased relative to the fiber-free control with fiber mixture 1 or fiber mixture 2, and numerically increased with fiber mixture 3, with increases observed as early as 6 h after supplementation (Figures 2A,B). Tapioca dextrin induced a numeric increase in acetate and a significant increase in propionate production relative to the fiber-free control. Levels of butyrate were numerically increased with all test products relative to the fiber-free control in the time interval of 6–24 h after supplementation (Figure 2C). A numeric increase in lactate production relative to the fiber-free control was also observed for all test products during the 0–6 h incubation period, which decreased at the later timepoints (Figure 2D). Measures of proteolytic fermentation, BCFA, and ammonium were numerically and significantly decreased, respectively, with all test products relative to the fiber-free control (Figures 2E,F). Data for individual donors are shown in Supplementary Figure S1. Donor to donor variations in SCFA production were observed; Donor A produced lower levels of butyrate relative to the other two donors.

### 3.2 Effects on microbial community composition

**Figure 3:**
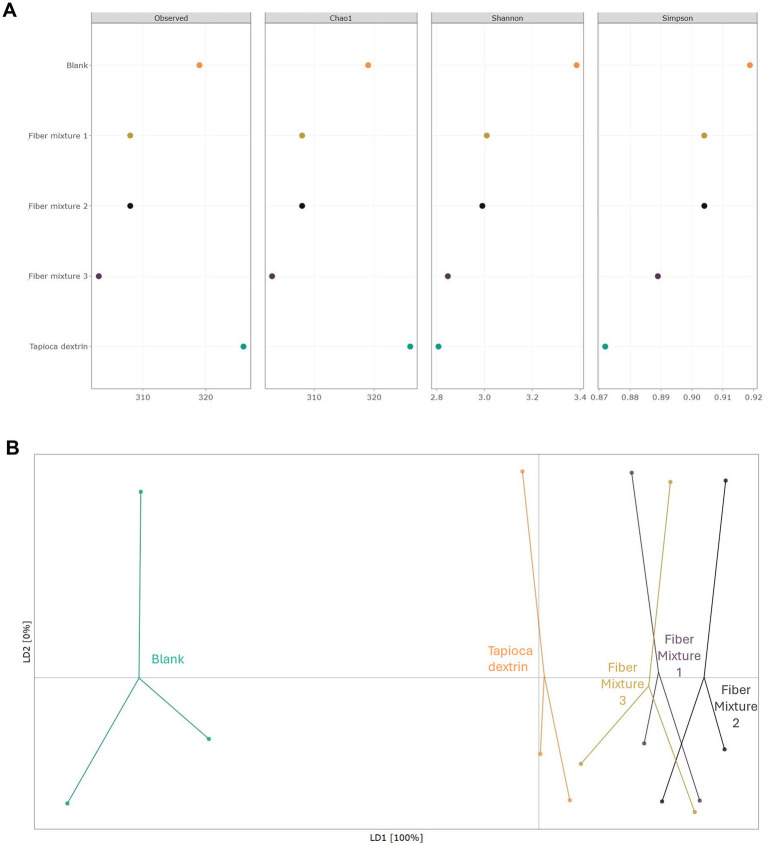
Analysis of the effects of fiber mixtures and tapioca dextrin 48 h after the start of the short-term colonic incubations compared to the fiber-free control (blank) (average across donors). **(A)** *α*-Diversity, represented by the observed, Chao1, Shannon, and Simpson diversity indices, based on relative abundance data (total sum scaling), and **(B)** *β*-diversity represented by a scatter plot. Each color represents a different treatment, and each dot represents a different donor. *n* = three donors.

Bacterial species richness (measured using the Observed and Chao1 indices) was decreased with the fiber mixtures and increased with tapioca dextrin relative to the fiber-free control (Figure 3A), though not reaching statistical significance. Bacterial species evenness (measured by the Shannon and Simpson indices) was reduced compared with the fiber-free control with all test products, reaching significance for fiber mixture 3 (Shannon: *p* = 0.0426) and tapioca dextrin (Shannon indices: *p* = 0.0295; Simpson indices: *p* = 0.0270). Furthermore, treatment with the fiber mixtures and tapioca dextrin significantly impacted community composition (*β*-diversity), as indicated by their segregation from the fiber-free control in Figure 3B. Fiber mixtures 1, 2, and 3 were found to share some similarities in community composition that were slightly distinct from tapioca dextrin, even though differences from the fiber-free control were more pronounced.

**Figure 4:**
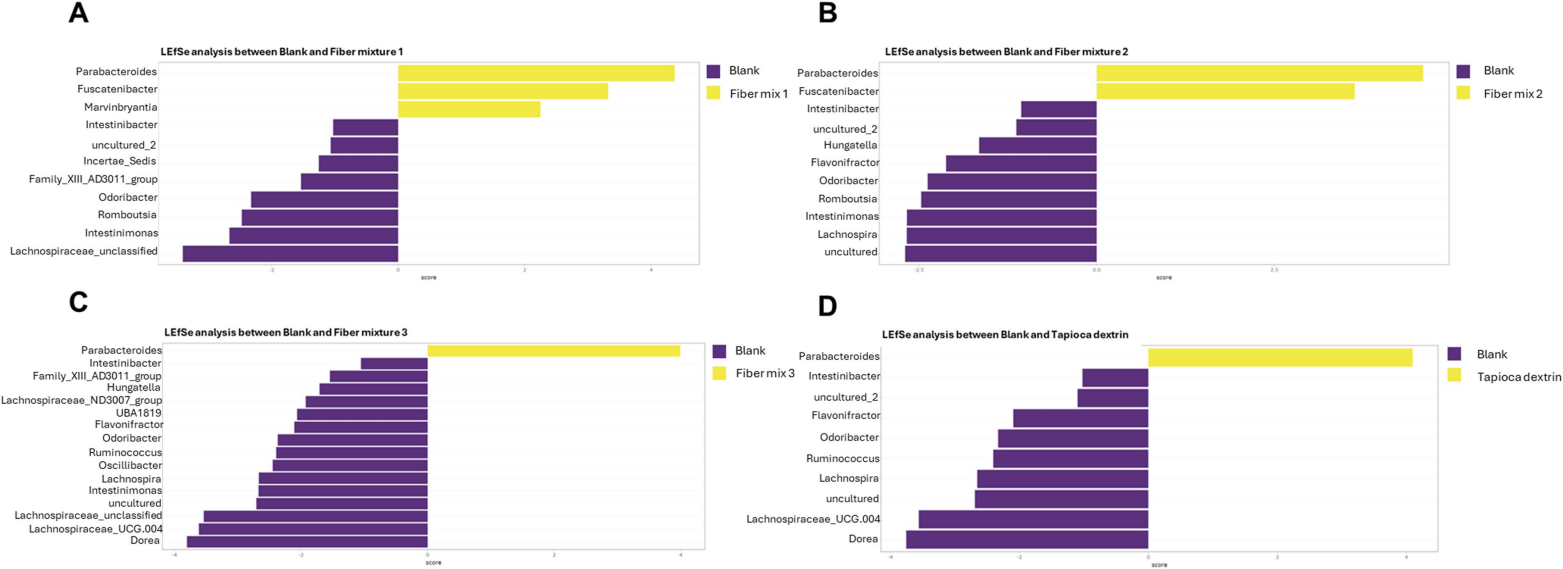
Differential abundance analysis (LEfSe) to identify differences in community composition at 24 h after the start of incubation with **(A)** fiber mixture 1, **(B)** fiber mixture 2, **(C)** fiber mixture 3, or **(D)** tapioca dextrin vs. the fiber-free control (blank). The analyses are based on relative abundance data (total sum scaling). The LEfSe bar plot shows significantly altered bacterial communities between the fiber-free control (blank) and the indicated test product. Sections highlighted in yellow represent features that were significantly enriched by the test product, while sections in purple were more abundant in the fiber-free control (blank). The *x*-axis represents the LDA score (measure of effect size), with LDA scores of ± 2 (generally accepted as biologically relevant) indicated by dotted lines. LDA, linear discriminant analysis; LEfSe, linear discriminant analysis effect size.

TreeclimbR analysis demonstrated a biologically and statistically significant increase in the abundance of the genera *Parabacteroides* (Bacteroidetes), *Fusicatenibacter* (Firmicutes), and unclassified *Bacteroidales* (Bacteroidetes) with all three fiber mixtures relative to the fiber-free control (Supplementary Figures S2A–C). The *Parabacteroides* increase was confirmed in LEfSe analysis for all three fiber mixtures, and the *Fusicatenibacter* was confirmed for fiber mixes 1 and 2 (Figures 4A–C). Biologically significant increases that were common to all three fiber mixes included *Holdemanella* (Firmicutes) and *Streptococcus* (Firmicutes). In addition to the common changes, fiber mixture 1 induced a biologically significant increase in *Blautia* (Firmicutes) (Supplementary Figure S2A). Fiber mixture 2 also stimulated a biologically and statistically significant increase in *Marvinbryantia* (Firmicutes) and a biologically significant increase in *Blautia*, *Erysipelotrichaceae* (family) (Firmicutes), and *Prevotellaceae* (family) (Bacteroidetes) (Figures 4B; Supplementary Figure S2B). Biologically significant increases unique to fiber mixture 3 were *Dialister* (Firmicutes), *Collinsella* (Actinobacteria), and *Bifidobacterium* (Actinobacteria) (Supplementary Figure S2C). Tapioca dextrin induced biologically and statistically significant increases vs. the fiber-free control for *Parabacteroides* and *Fusicatenibacter* and biologically significant increases in *Bacteroidales*, *Bifidobacterium*, *Streptococcus*, and *Prevotellaceae* (family); the increase in *Parabacteroides* was also observed with LEfSe analysis (Figures 4D; Supplementary Figure S2D).

### 3.3 Specific modulation of *Bifidobacterium*, Firmicutes, and Bacteroidetes based on qPCR quantification

**Figure 5:**
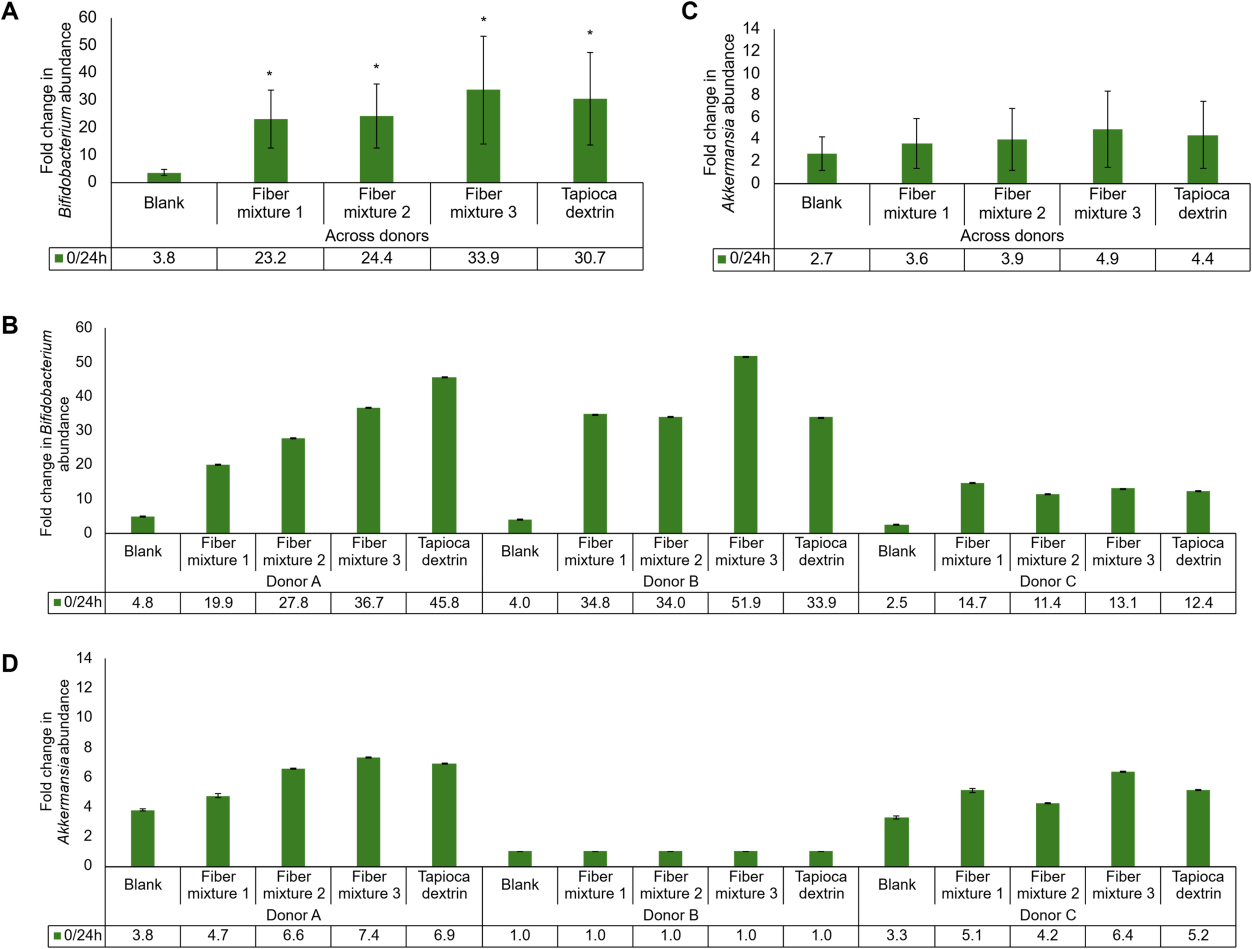
Fold change in average abundance of *Bifidobacterium* **(A)** across donors or **(B)** for individual donors and *Akkermansia* **(C)** across donors or **(D)** for individual donors from 0 h to 24 h. Paired two-sided *t*-tests were used to determine significant differences between the test product and the fiber-free control (blank). A *p*-value of <0.05 was considered statistically significant.

At both 6 and 24 h, the FC in *Bifidobacterium* abundance from baseline (0 h) was significantly greater with all three fiber mixtures and tapioca dextrin relative to the fiber-free control across donors (Figures 5A; Supplementary Figure S3A). When looking at individual donors, the FC in *Bifidobacterium* abundance with all test products relative to the fiber-free control was high for Donors A and B, while the effect was less pronounced for Donor C at both timepoints (Figures 5B; Supplementary Figure S3B). There were no significant differences for any of the test products relative to the fiber-free control for FC in *Akkermansia* abundance from baseline to 6 h or 24 h (Figures 5C; Supplementary Figure S3C). There was a high level of variability in FC for *Akkermansia* abundance among donors, with Donor A having the greatest effect, followed by Donor C, and very little change in any condition for Donor B (Figures 5D; Supplementary Figure S3D). However, improved *Akkermansia* growth was observed at 24 h for all test products for Donors A and C. Across donors, FC was significantly higher from baseline in Firmicutes and Bacteroidetes with fiber mixtures 1 and 2 and with tapioca dextrin compared with the fiber-free control at 6 h and with all test products at 24 h (Supplementary Figures S4A–C). When examining individual donors, interindividual differences in response were minimal (Supplementary Figures S4B–D).

### 3.4 Effects on inflammation-induced damage to the intestinal epithelium and cytokine production

**Figure 6:**
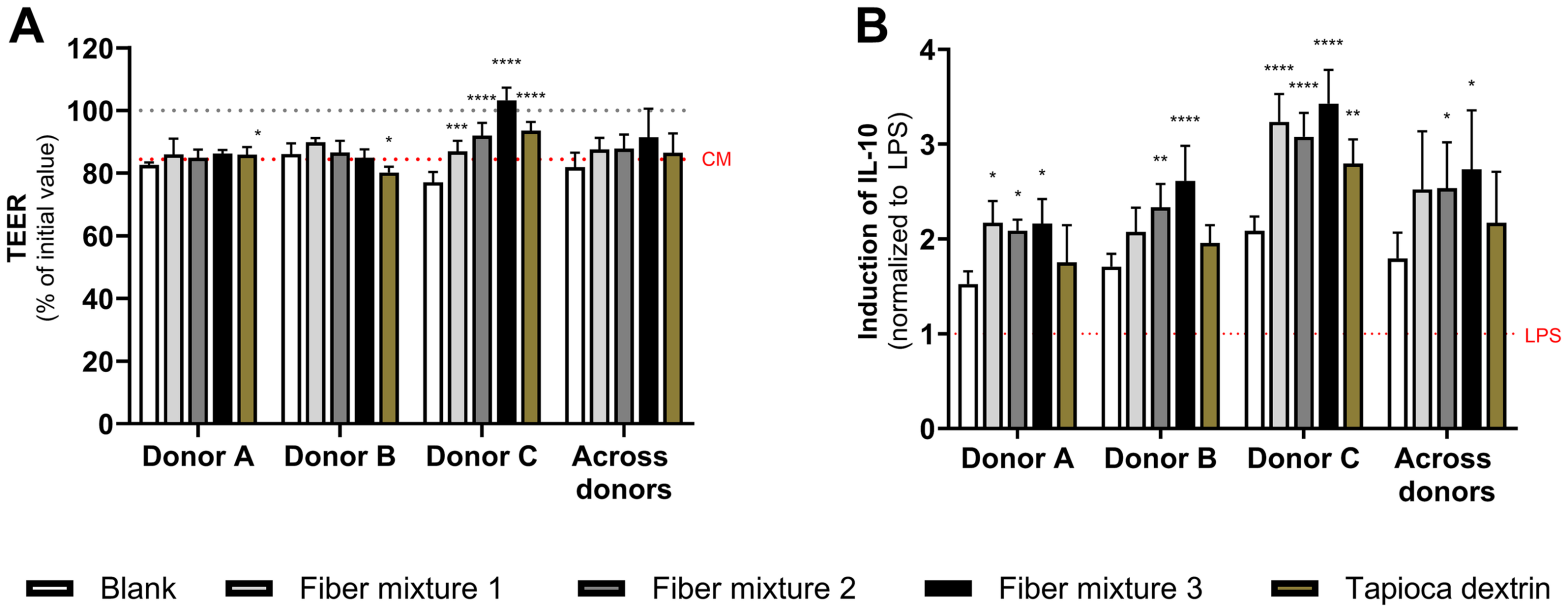
Effect of colonic suspensions following fermentation of fiber mixtures or tapioca dextrin on the **(A)** epithelial cell barrier integrity (TEER) and **(B)** IL-10 after LPS stimulation in the Caco-2/THP1 co-culture model. Data are plotted as mean ± standard deviation. In panel **(A)**, the grey dotted line represents 100% (initial value), and the red dotted line corresponds to the experimental control complete medium (CM). For panel **(B)**, each value was normalized to the average of the LPS control; the red dotted line corresponds to the experimental control LPS. To assess differences in TEER and IL-10 between the test products and fiber-free control (blank) for individual donors, a two-way ANOVA with Dunnett’s multiple comparisons test against the fiber-free control was used, and for the average of the three donors, a paired two-tailed *t*-test was performed, using the average of the technical replicates of the individual donors as input values. *p < 0.05, **p < 0.01, ***p < 0.001, ****p < 0.0001. CM, complete medium; IL, interleukin; LPS, lipopolysaccharide; TEER, transepithelial electrical resistance.

For all treatments, donor-dependent effects on the intestinal epithelial barrier integrity were observed. Treatment with all test product fermentations significantly increased TEER in Donor C compared to their respective fiber-free control. Therefore, TEER values indicated that fiber mixtures and tapioca dextrin fermentations provided significant protection against inflammation-induced barrier disruption relative to fiber-free fermentations in Donor C (Figure 6A). No significant protection was observed with any of the fiber mixtures for Donors A and B; there was a significant decrease in protection with tapioca dextrin for Donor B. Following stimulation with LPS, anti-inflammatory IL-10 was greatly induced by test product fermentations, which reached significance for Donors A and C with fiber mixture 1, across donors with fiber mixtures 2 and 3, and for Donor C with tapioca dextrin (Figure 6B). NF-κB activity was increased with fiber mixture 1 (Donor B and C, across donors), fiber mixture 2 (Donor A and C, across donors), and fiber mixture 3 (across donors) but not with tapioca dextrin (Supplementary Figure S5A). The test product fermentations had little effect on IL-6, IL-1β, TNF-α, CXCL10, or IL-8 levels (Supplementary Figures S5B–F).

## 4 Discussion

This study confirmed the beneficial physiological effects and prebiotic potential of three dietary fiber blends, each containing chicory inulin and wheat dextrin in varying proportions, with two mixtures incorporating cellulose and one without, in an *in vitro* colonic simulation model, compared to the established prebiotic tapioca dextrin. All test products were efficiently fermented by the fecal microbiota from three healthy donors, confirming their resistance to digestion in the upper gastrointestinal tract. Fermentation resulted in SCFA production, including butyrate, and modulated the gut microbial community by increasing saccharolytic bacteria, including those in the phyla Actinobacteria, Bacteroidetes, and Firmicutes. Simultaneously, proteolytic fermentation markers, that is, ammonium, were significantly reduced, underscoring the health-promoting potential of these fibers. Moreover, the mixtures showed anti-inflammatory and immunomodulatory effects contributing to the protection of gut barrier function.

Dietary fiber is legally defined in EU Directive 2008/100/EC as edible carbohydrate polymers with at least three monomeric units that escape digestion and absorption in the small intestine and exert physiological benefits (12). While fermentability by gut microbiota is one such mechanism, it is not a prerequisite. Prebiotics represent a functionally distinct subgroup—often within dietary fibers—that must resist digestion, be fermented by the gut microbiota, and selectively enhance microbial populations with positive effects on host health (13, 49). *In vitro* models, such as short-term colonic simulations, provide controlled conditions to study these effects (50–52). The model used in our study relies on establishing representative fecal microbial communities from human volunteers (50, 53–55).

The fiber mixtures and tapioca dextrin were predigested to mimic upper gastrointestinal passage, as they include digestible compounds that would otherwise be absorbed in the small intestine. Their subsequent efficient fermentation in the colonic simulation model shows they can withstand stomach acidity and mammalian enzyme hydrolysis. Fermentation in the colonic model decreased pH and increased gas production, and elevated SCFAs levels over 48 h compared to the fiber-free control. While butyrate production was stimulated in two of the three donors, interindividual variability prevented statistical significance. Lactate depletion after 24 h suggested cross-feeding mechanisms, in which lactate-utilizing bacteria probably contributed to butyrate production, indirectly enhancing the growth of health-associated butyrate producers (20, 56–58).

Proteolytic fermentation markers, including ammonium and BCFAs, were reduced compared to the fiber-free control. Toxic by-products of proteolytic fermentation are linked to intestinal inflammation, barrier dysfunction, and systemic diseases, including chronic kidney disease and colorectal cancer. Lowered levels of these metabolites highlight the potential for these fibers to mitigate gut and systemic health risks (59). Proteolytic fermentation in the colon generates an array of potentially toxic by-products, including ammonium, amines, hydrogen sulfide, phenols, indoles, and para-cresol (59). Increased gut ammonium concentrations contribute to intestinal barrier dysfunction and increased inflammation (60, 61). Para-cresol is derived from the metabolism of the aromatic amino acids tyrosine and phenylalanine and is known for its role as a uremic toxin. It can induce DNA damage leading to local and systemic detrimental effects such as decreased colonocyte proliferation, renal tubular damage, and endothelial dysfunction (59, 62). Clinically, increased levels of gut-derived uremic toxins can contribute to inflammation, fibrosis, endocrine, metabolic, and neurologic disorders, protein-energy wasting, and the progression of chronic kidney disease (60, 63).

Another characteristic feature of prebiotic fibers is the ability to shift the microbial composition in the colon. All fiber mixtures and tapioca dextrin demonstrated this feature by increasing *Bifidobacterium*, *Collinsella*, *Parabacteroides*, and *Fusicatenibacter*, which are known for SCFA production, anti-inflammatory properties, and pathogen exclusion (56, 64–74). Fiber mixture 3, with over 80% inulin and no cellulose, had the most pronounced impact on microbial activity and composition, likely due to cellulose’s limited fermentation capacity (75). Fiber mixture 3 stimulated biologically significant increases in acetate- and lactate-producing *Bifidobacterium* and *Collinsella* spp., and lactate-producing *Streptococcus* spp. (56, 64, 65). *Collinsella* spp. can also influence metabolism by decreasing hepatic glycogenesis, altering intestinal cholesterol absorption, and increasing triglyceride synthesis (66). *Parabacteroides*, which degrade complex polysaccharides, regulate the host immune system, and produce acetate and succinate (67), were also increased, along with *Dialister*, which produce health-promoting propionate from succinate (68). The increase of these two genera could indicate the possibility of succinate cross-feeding. *Fusicatenibacter* and *Holdemanella*, which can produce acetate, lactate, and butyrate (69, 70), were also increased with supplementation. Fiber mixture 2, with the highest proportion of wheat dextrin, had the strongest propiogenic effect, which was likely linked to the strong enrichment of *Parabacteroides* spp., *Fusicatenibacter*, *Marvinbryantia*, *Blautia*, *Streptococcus*, *Holdemanella*, and *Prevotellaceae* spp. were also enriched with fiber mixture 2. Each of these genera produces a variety of SCFAs (56, 69–73). Additional health-promoting activities of these genera include anti-inflammatory effects in *Fusicatenibacter* (74) and the potential to support metabolic health in *Blautia* (76).

Colonic fermentation of each of the fiber mixtures and tapioca dextrin resulted in protection of the intestinal epithelial barrier from inflammation-induced damage in one of the three donors, as indicated by significantly increased TEER values. This is of interest, as lower TEER values indicate permeability in the epithelial barrier, which is associated with leaky gut (77). Additionally, immunomodulatory effects were observed. The anti-inflammatory cytokine IL-10 was significantly increased with all fiber mixtures and in all donors (except Donor B) with fiber mixture 1. IL-10 production was also significantly increased in Donor C with tapioca dextrin. IL-10 helps control inflammatory responses in the gut (78). It is associated with improved colitis and is thought to limit colitis-associated colorectal cancer (79). The increased production of health-promoting SCFAs, improved intestinal epithelial barrier function following an inflammatory insult (in Donor C), and increased production of the anti-inflammatory cytokine IL-10 support the fiber mixtures’ potential to improve the host’s overall health.

The results of this study demonstrate that the tested fiber blends fulfil key dietary fiber criteria and exhibit beneficial physiological effects, making them suitable for inclusion in enteral nutrition formulations. The ability to increase SCFA production, reduce proteolytic fermentation, and modulate gut microbiota supports their role in maintaining gut homeostasis. The observed immunomodulatory and barrier-protective effects further underscore their potential to mitigate the challenges posed by gut dysbiosis and inflammation commonly encountered in patients receiving enteral nutrition, including those who are critically ill, have gastrointestinal disorders, or are immunocompromised. Modulation of the gut microbiota through dietary fibers is a promising approach for improving human health across a broad range of conditions and diseases (80). Nutritional interventions, such as increased dietary fiber intake, can alter the composition and metabolic activity of the gut microbiome, potentially leading to health benefits (3). Different types of dietary fiber differentially influence gut microbiota composition and metabolic status (81). The prebiotic effects of inulin and wheat dextrin have been previously characterized. Inulin efficiently produces SCFAs, particularly butyrate, as confirmed in *in vitro* fermentation models (82, 83). Data from human intervention trials in patients with intestinal dysfunction or at risk of critical illness showed that inulin restores gut microbial balance (84). The prebiotic effect of wheat dextrin starch depends on its structure and source, but overall evidence shows growth of *Lactobacillus* spp. and prebiotic effects resulting from bacterial fermentation (27, 85). Cellulose, though a bulking fiber and not a classical prebiotic given its limited fermentation, has also been reported to alter the gut microbiota, impact colonic epithelial cell gene expression, and affect intestinal barrier function (75). Even though we found some combination-specific and donor-specific variations, our study demonstrates that all three dietary fiber mixtures tested, as well as tapioca dextrin, exhibit beneficial prebiotic properties, confirming the results obtained for the single components.

We acknowledge that the study has some limitations. The number of donors may have been too small to account for interindividual differences and to generalize these findings to the broader public. Therefore, the study design was adapted accordingly, by focusing on specific fermentation parameters (SCFA, lactate, ammonium) that are generally produced by healthy persons, irrespective of differences in community composition due to functional redundancy, and by adjusting the level of depth (i.e., the taxonomic resolution) with which changes in community composition have been discussed (genus level rather than species level). In addition, *in vitro* studies cannot fully replicate a test product’s effects on complex human physiological processes. Nevertheless, short-term colonic simulations generate data on how test products interact with the human gut microbiota, including microbial composition and activity. Along this line, Perreau et al. (86) recently reported the effectiveness of short-term colonic simulations. Their study revealed that the reported effects of the prebiotic fiber NUTRIOSE® on gut microbiota activity and composition were robustly correlated between clinical trial studies and studies using a Colon-on-a-plate® model (miniaturization of the short-term colonic simulation). This underscores the value of this methodology in mimicking *in vivo* conditions and informing test product development for clinical trials.

## 5 Conclusion

Using a clinically relevant *in vitro* model, we demonstrated that dietary fiber mixtures containing different proportions of inulin, wheat dextrin, and cellulose exhibit prebiotic properties. These include stimulating SCFA production and promoting the growth of beneficial bacteria such as *Bifidobacterium*. The prebiotic tapioca dextrin also showed similar properties. All tested fiber mixtures retained their prebiotic properties when combined, demonstrating effectiveness across the tested ratios. Given their positive impact on colonic microbiota composition and activity, these fiber mixtures are expected to benefit not only healthy individuals but also patients at risk of gut dysbiosis. The findings support the incorporation of these fiber blends into enteral tube feeds or oral nutritional supplements for patients requiring Medical Nutrition Therapy, a finding that should be confirmed in future studies. Overall, this study provides evidence that these mixtures confer physiological benefits that promote gut health and support overall well-being in diverse populations.

## Data Availability

The data analyzed in this study was obtained from ProDigest, the following licenses/restrictions apply: IP restrictions with respect to used methods. Requests to access these datasets should be directed to the corresponding author, Massimo Marzorati (massimo.marzorati@prodigest.eu).
