# Supplementary material for: Fiber mixtures containing chicory inulin, wheat dextrin, and cellulose, or tapioca dextrin alone, beneficially modulate microbial metabolic activity and composition in short-term colonic simulations
Source: Front Nutr. 2026 Feb 18;13:1749272. doi: 10.3389/fnut.2026.1749272 (PMC12957075; doi:10.3389/fnut.2026.1749272)
Supplement: Supplementary file 1 [file Supplementary_file_1.pdf]

Supplementary Material

1 Supplementary Figures

A

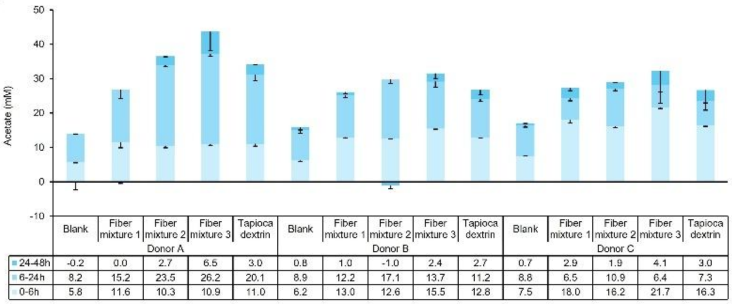

	Blank	Fiber mixture 1	Fiber mixture 2	Fiber mixture 3	Tapioca dextrin	Blank	Fiber mixture 1	Fiber mixture 2	Fiber mixture 3	Tapioca dextrin	Blank	Fiber mixture 1	Fiber mixture 2	Fiber mixture 3	Tapioca dextrin
	Donor A					Donor B					Donor C				
24-48h	-0.2	0.0	2.7	6.5	3.0	0.8	1.0	-1.0	2.4	2.7	0.7	2.9	1.9	4.1	3.0
6-24h	8.2	15.2	23.5	26.2	20.1	8.9	12.2	17.1	13.7	11.2	8.8	6.5	10.9	6.4	7.3
0-6h	5.8	11.6	10.3	10.9	11.0	6.2	13.0	12.6	15.5	12.8	7.5	18.0	16.2	21.7	16.3

B

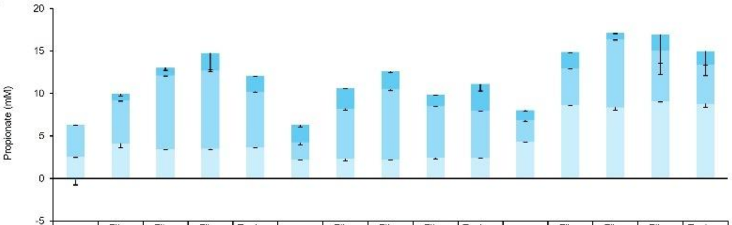

	Blank	Fiber mixture 1	Fiber mixture 2	Fiber mixture 3	Tapioca dextrin	Blank	Fiber mixture 1	Fiber mixture 2	Fiber mixture 3	Tapioca dextrin	Blank	Fiber mixture 1	Fiber mixture 2	Fiber mixture 3	Tapioca dextrin
	Donor A					Donor B					Donor C				
24-48h	-0.1	0.8	0.9	2.1	1.8	2.0	2.4	2.1	1.4	3.1	1.2	1.9	0.7	1.9	1.6
6-24h	3.8	5.0	8.7	9.2	6.6	2.1	5.9	8.3	6.1	5.6	2.5	4.3	8.0	6.0	4.6
0-6h	2.6	4.1	3.4	3.5	3.7	2.2	2.3	2.2	2.4	2.4	4.3	8.6	8.4	9.1	8.8

C

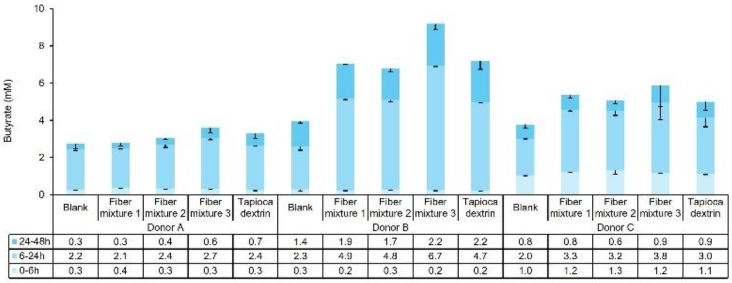

	Blank	Fiber mixture 1	Fiber mixture 2	Fiber mixture 3	Tapioca dextrin	Blank	Fiber mixture 1	Fiber mixture 2	Fiber mixture 3	Tapioca dextrin	Blank	Fiber mixture 1	Fiber mixture 2	Fiber mixture 3	Tapioca dextrin
	Donor A					Donor B					Donor C				
24-48h	0.3	0.3	0.4	0.6	0.7	1.4	1.9	1.7	2.2	2.2	0.8	0.8	0.6	0.9	0.9
6-24h	2.2	2.1	2.4	2.7	2.4	2.3	4.9	4.8	6.7	4.7	2.0	3.3	3.2	3.8	3.0
0-6h	0.3	0.4	0.3	0.3	0.3	0.3	0.2	0.3	0.2	0.2	1.0	1.2	1.3	1.2	1.1

D

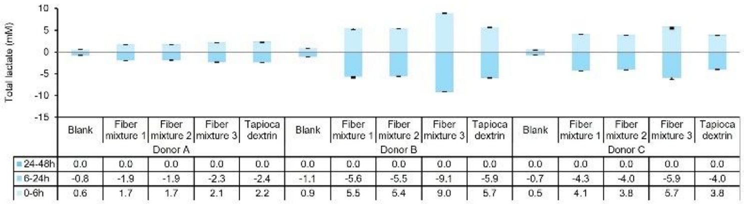

	Blank	Fiber mixture 1	Fiber mixture 2	Fiber mixture 3	Tapioca dextrin	Blank	Fiber mixture 1	Fiber mixture 2	Fiber mixture 3	Tapioca dextrin	Blank	Fiber mixture 1	Fiber mixture 2	Fiber mixture 3	Tapioca dextrin
	Donor A					Donor B					Donor C				
24-48h	0.0	0.0	0.0	0.0	0.0	0.0	0.0	0.0	0.0	0.0	0.0	0.0	0.0	0.0	0.0
6-24h	-0.8	-1.9	-1.9	-2.3	-2.4	-1.1	-5.6	-5.5	-9.1	-5.9	-0.7	-4.3	-4.0	-5.9	-4.0
0-6h	0.6	1.7	1.7	2.1	2.2	0.9	5.5	5.4	9.0	5.7	0.5	4.1	3.8	5.7	3.8

E

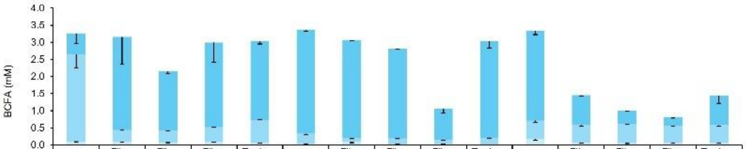

	Blank	Fiber mixture 1	Fiber mixture 2	Fiber mixture 3	Tapioca dextrin	Blank	Fiber mixture 1	Fiber mixture 2	Fiber mixture 3	Tapioca dextrin	Blank	Fiber mixture 1	Fiber mixture 2	Fiber mixture 3	Tapioca dextrin
	Donor A					Donor B					Donor C				
24-48h	0.6	2.7	1.7	2.5	2.3	3.0	2.9	2.6	0.9	2.8	2.6	0.9	0.4	0.3	0.9
6-24h	2.6	0.4	0.4	0.4	0.7	0.3	0.1	0.2	0.1	0.2	0.5	0.5	0.6	0.5	0.5
0-6h	0.1	0.1	0.1	0.1	0.1	0.0	0.1	0.0	0.0	0.0	0.2	0.1	0.1	0.1	0.1

F

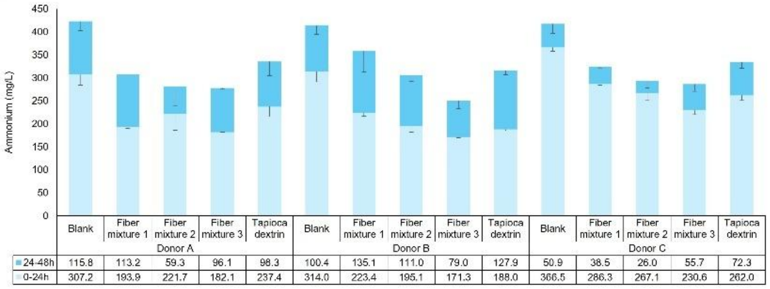

	Blank	Fiber mixture 1	Fiber mixture 2	Fiber mixture 3	Tapioca dextrin	Blank	Fiber mixture 1	Fiber mixture 2	Fiber mixture 3	Tapioca dextrin	Blank	Fiber mixture 1	Fiber mixture 2	Fiber mixture 3	Tapioca dextrin
	Donor A					Donor B					Donor C				
24-48h	115.8	113.2	59.3	96.1	98.3	100.4	135.1	111.0	79.0	127.9	50.9	38.5	26.0	55.7	72.3
0-24h	307.2	193.9	221.7	182.1	237.4	314.0	223.4	195.1	171.3	188.0	366.5	286.3	267.1	230.6	262.0

Supplementary Figure S1. Effect of fiber mixtures and tapioca dextrin on **(A)** acetate (mM), **(B)** propionate (mM), **(C)** butyrate (mM), **(D)** lactate (mM), **(E)** branched chain fatty acids (BCFA; mM), and **(F)** ammonium (mg/L) at different time intervals. Each condition was tested in duplicate and values are shown for each of three healthy donors. Error bars represent standard deviation. BCFA, branched chain fatty acid.

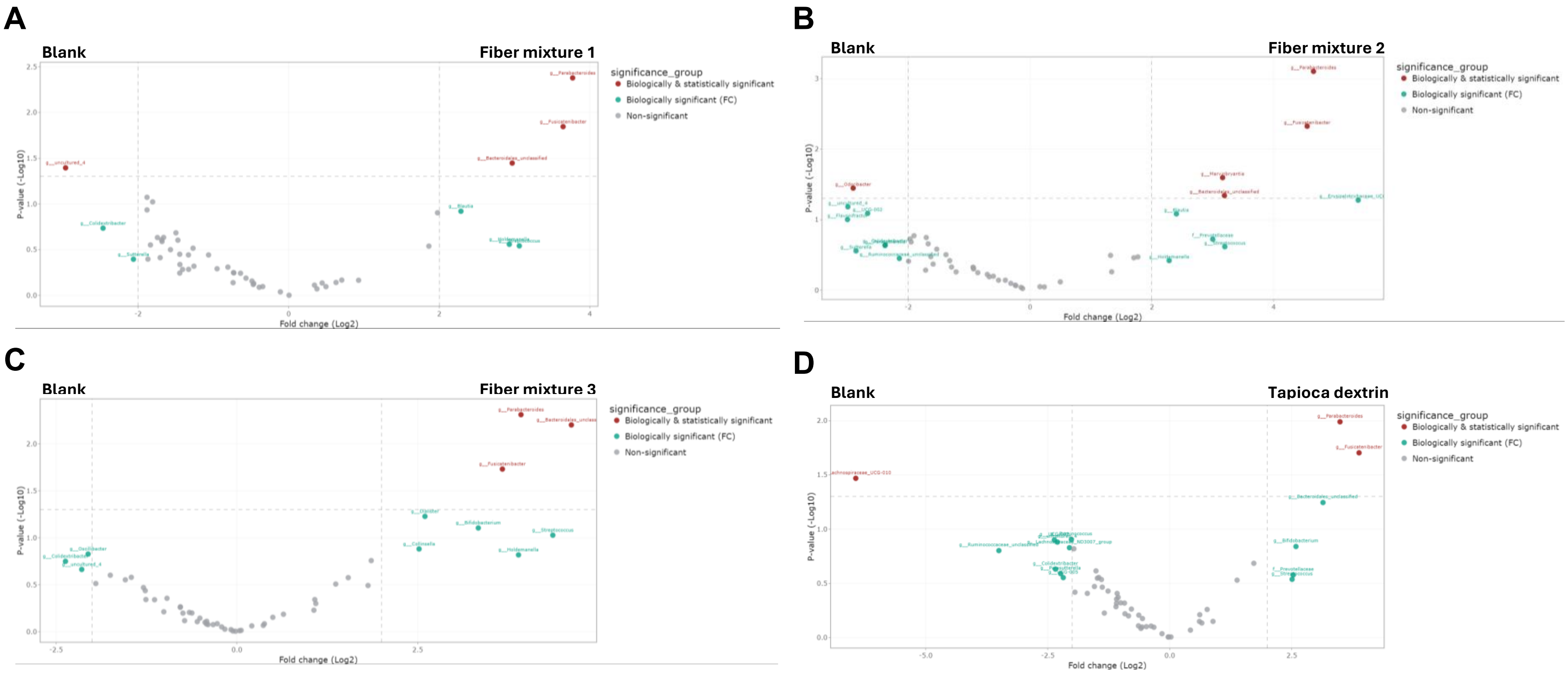

Supplementary Figure S2. Differential abundance analysis (treeclimbR) to identify differences in community composition at 24 h after the start of incubation with (A) fiber mixture 1, (B) fiber mixture 2, (C) fiber mixture 3, or (D) tapioca dextrin versus the fiber-free control (blank). The analyses are based on relative abundance data (total sum scaling). The scatter plot classifies taxa into four categories based on abundances in the compared conditions: neither biologically or statistically significant (gray), biologically significant but not statistically significant (teal), statistically significant but not biologically significant (blue), or biologically and statistically significant (red).

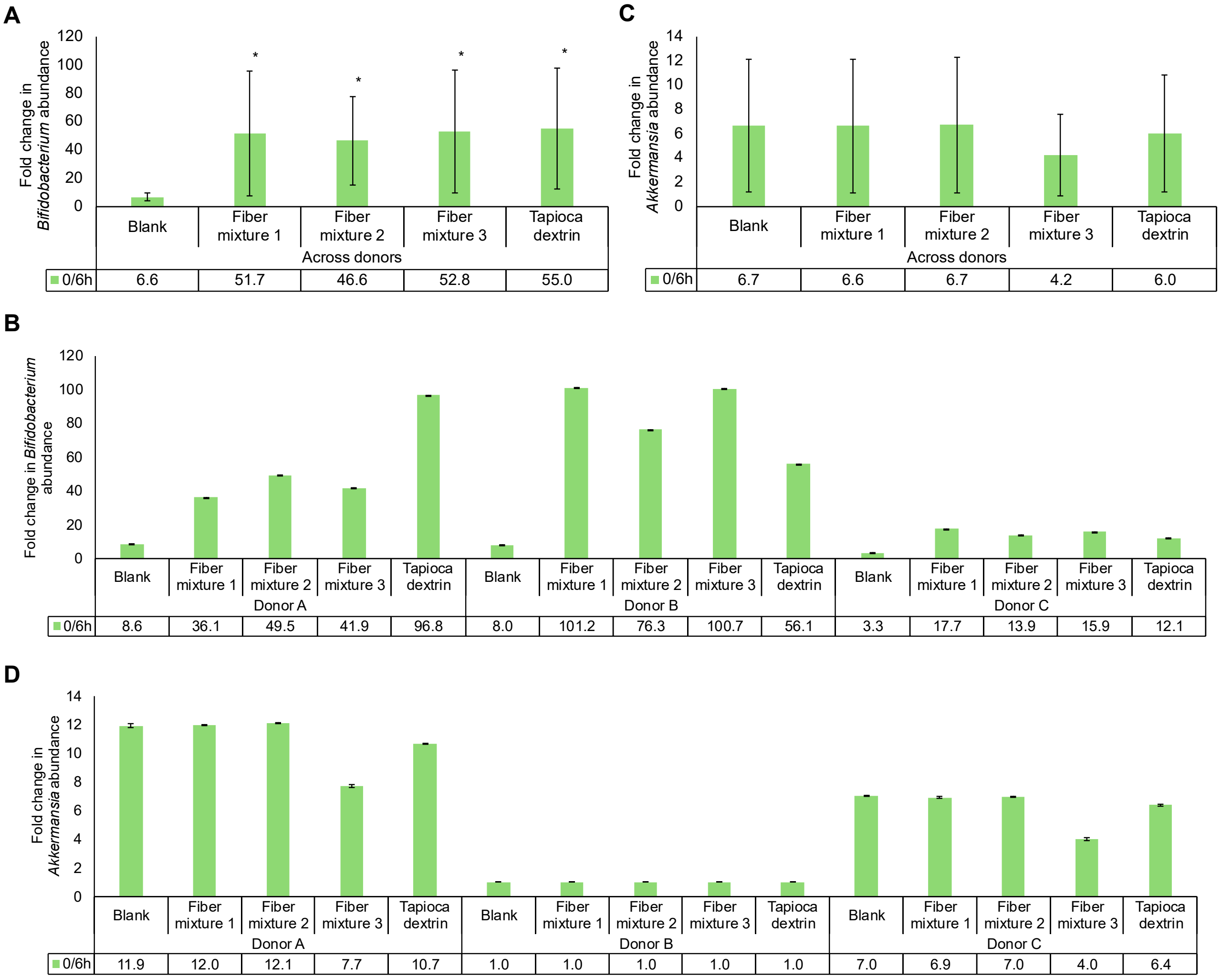

Supplementary Figure S3. Fold change in average abundance of *Bifidobacterium* (A) across donors or (B) for individual donors and *Akkermansia* (C) across donors or (D) for individual donors from 0 h to 6 h. Paired two-sided t-tests were used to determine significant differences between test product and the fiber-free control (blank). A p-value of <0.05 was considered statistically significant.

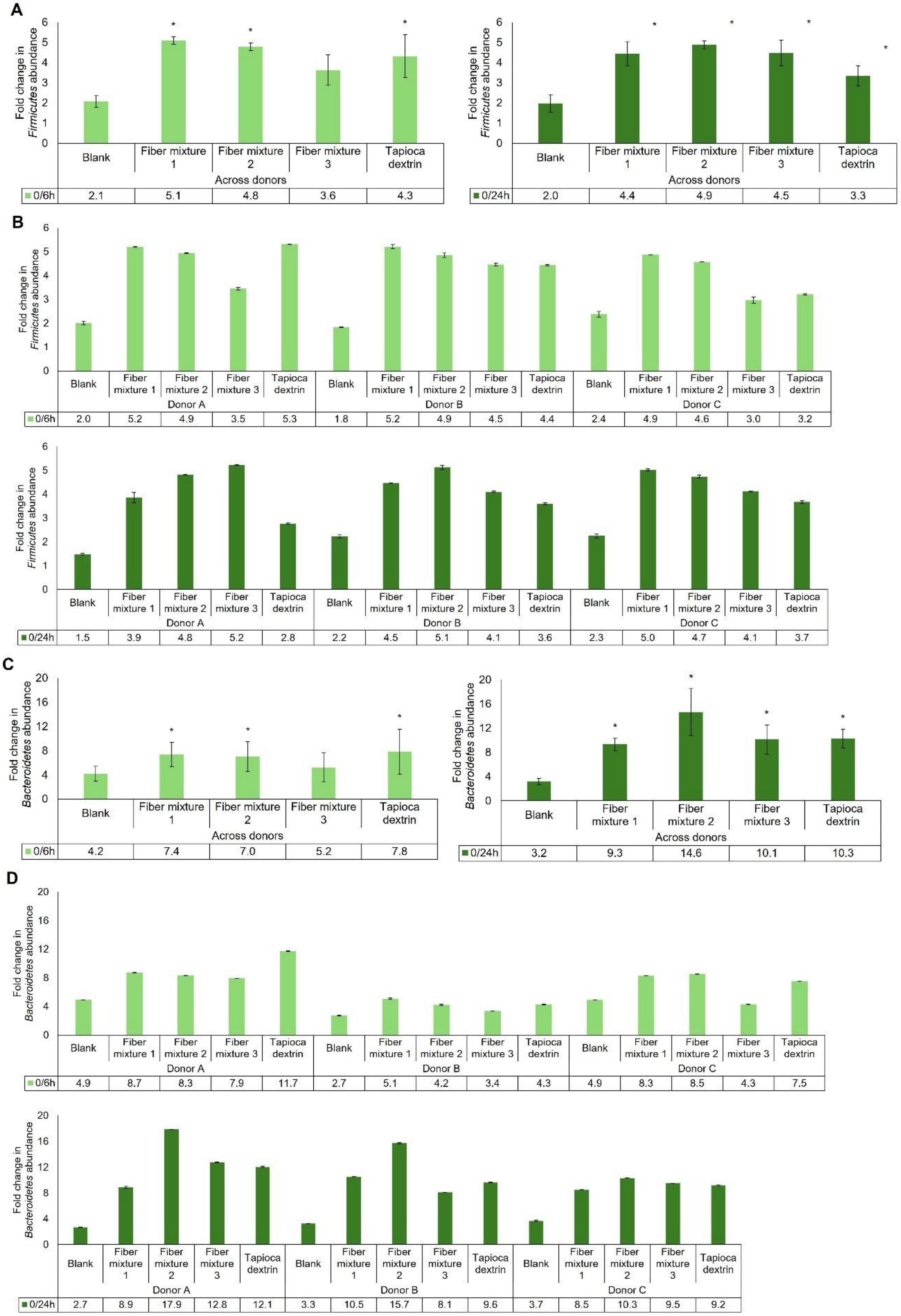
A
Fold change in Firmicutes abundance
Blank
Fiber mixture 1
Fiber mixture 2
Fiber mixture 3
Tapioca dextrin
Across donors
0/6h 2.1 5.1 4.8 3.6 4.3
0/24h 2.0 4.4 4.9 4.5 3.3
B
Fold change in Firmicutes abundance
Donor A
Donor B
Donor C
0/6h 2.0 5.2 4.9 3.5 5.3 1.8 5.2 4.9 4.5 4.4 2.4 4.9 4.6 3.0 3.2
0/24h 1.5 3.9 4.8 5.2 2.8 2.2 4.5 5.1 4.1 3.6 2.3 5.0 4.7 4.1 3.7
C
Fold change in Bacteroidetes abundance
Across donors
0/6h 4.2 7.4 7.0 5.2 7.8
0/24h 3.2 9.3 14.6 10.1 10.3
D
Fold change in Bacteroidetes abundance
Donor A
Donor B
Donor C
0/6h 4.9 8.7 8.3 7.9 11.7 2.7 5.1 4.2 3.4 4.3 4.9 8.3 8.5 4.3 7.5
0/24h 2.7 8.9 17.9 12.8 12.1 3.3 10.5 15.7 8.1 9.6 3.7 8.5 10.3 9.5 9.2

Supplementary Figure S4. Fold change in average abundance of Firmicutes **(A)** across donors or **(B)** for individual donors and Bacteroidetes **(C)** across donors or **(D)** for individual donors from 0 h to 6 h or 24 h. Paired two-sided t-tests were used to determine significant differences between test product and the fiber-free control (blank). A p-value of <0.05 was considered statistically significant.

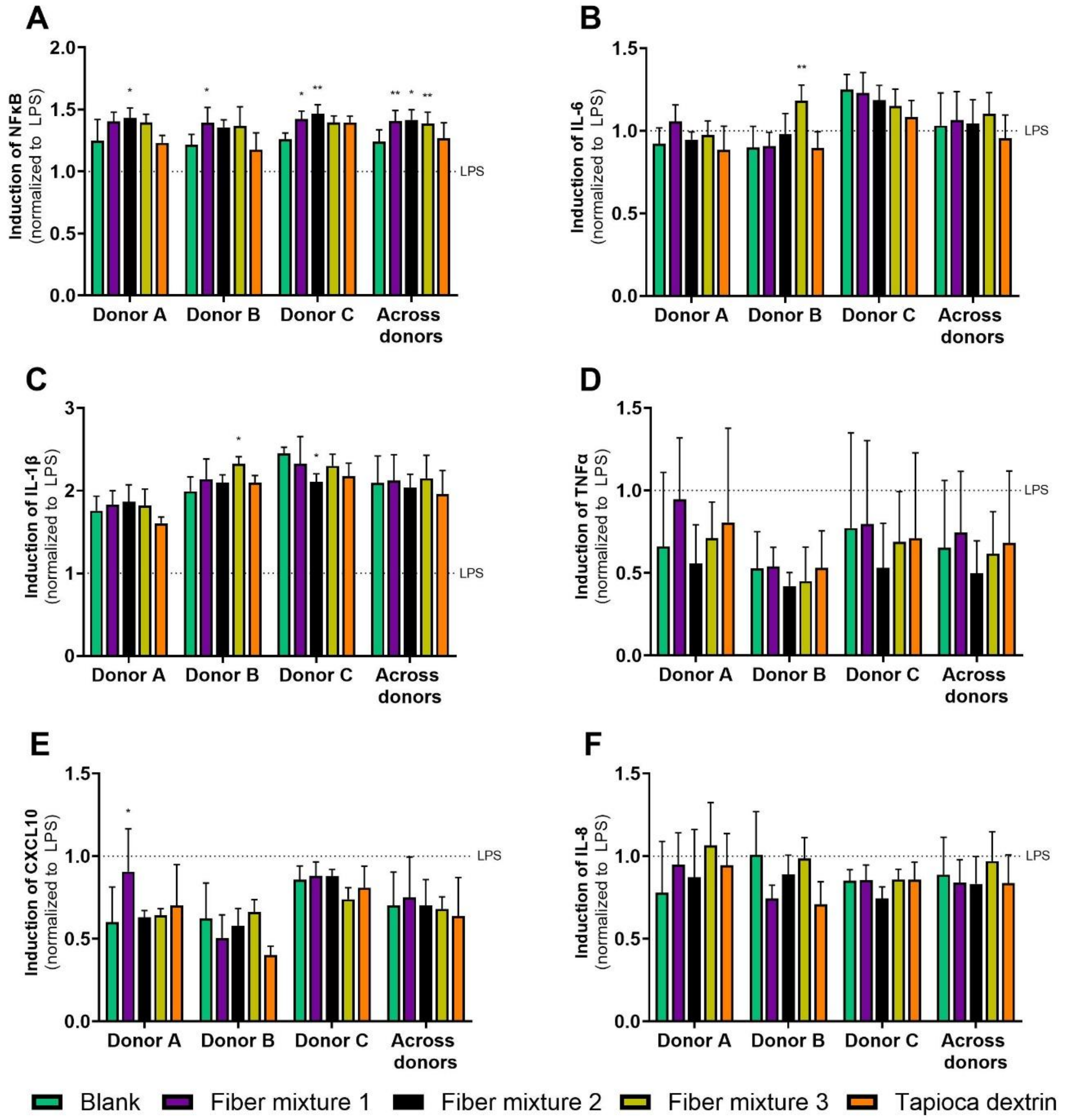

Supplementary Figure S5. Effect of colonic suspensions following fermentation of fiber mixtures or tapioca dextrin on the **(A)** NF-κB activity, or the secretion of **(B)** IL-6, **(C)** IL-1β, **(D)** TNFα, **(E)** CXCL10, or **(F)** IL-8 after LPS stimulation in the Caco-2/THP1 co-culture model. Data are plotted as mean ± standard deviation. Each value was normalized to the average of the LPS control; the black dotted line corresponds with the experimental control LPS. To assess differences in immune markers between the test products and the fiber-free control (blank) for individual donors, a two-way ANOVA with Dunnett's multiple comparisons test against the fiber-free control was used, and for the average of the three donors, paired two-tailed t-test were performed, using the average of the technical replicates of the individual donors as input values. * $p < 0.05$, ** $p < 0.01$, *** $p < 0.001$, **** $p < 0.0001$. IL, interleukin; LPS, lipopolysaccharide; TNF, tumor necrosis factor.
